# Dynamic photoelectron transport in stepwise-doped GaAs photocathodes

**DOI:** 10.1038/s41598-022-16993-8

**Published:** 2022-07-28

**Authors:** Rui Zhou, Hemang Jani, Yijun Zhang, Yunsheng Qian, Lingze Duan

**Affiliations:** 1 Department of Physics & Astronomy, The University of Alabama in Huntsville, Huntsville, 35899 USA; 2 School of Electronic and Optical Engineering, Nanjing University of Science and Technology, Nanjing, 210094 China

**Keywords:** Ultrafast photonics, Optoelectronic devices and components, Photonic devices

## Abstract

We present a theoretical model describing photoelectron transport dynamics in stepwise-doped GaAs photocathodes. Built-in electric field caused by the doping structure is analyzed, and the time-evolution of electron concentration in the active layer induced by a femtosecond laser pulse is solved. The predictions of the model show excellent agreement with the experimental data measured with pump-probe transient reflectometry, demonstrating the capability of the theoretical model in predicting photoelectron behaviors in real devices. Comparisons are also made between this stepwise doping model and the conventional gradient doping model with a continuous doping profile, thereby providing the first quantitative evaluation of the effectiveness and the limitation of the gradient doping model in describing actual stepwise-doped devices.

## Introduction

Semiconductor photocathodes with gradient doping structures (also known as exponential doping structures) have attracted lots of interest in recent years due to their improved performances over the conventional, uniform-doped devices, such as higher quantum efficiencies, longer diffusion lengths, and better spectral responses^1,2^. However, a true gradient-doping profile with continuously varying doping concentrations is very difficult to fabricate with the current manufacturing technology. The so-called “gradient-doped” devices are typically made up of multiple *uniformly* doped sublayers with a *stepwise* doping profile. On the other hand, it has been a common practice in the field to use continuous doping models to describe such stepwise-doped devices^3,4^, and this approximation has been widely adopted without any in-depth study on its validity. Due to the intrinsic difference between a continuous doping profile and a stepwise doping profile, certain important characteristics of real devices are missing in a simplified model based on a continuous doping profile. For example, the built-in electric field induced by a stepwise doping profile only appears within narrow regions across the sublayer boundaries rather than uniformly distributed throughout the active layer of the device. This leads to a different electron-population distribution inside the active layer and ultimately affects electron transport. The potential impact of such a difference has been pointed out^5^, but has not been carefully studied in the past.

Here, we present a theoretical model specifically developed to analyze the dynamic transport of photoelectrons following a femtosecond pulse excitation in a stepwise-doped GaAs photocathode. This work is built upon our previous studies of GaAs photocathodes with uniform-doping profiles^6^ and continuous gradient-doping profiles^5,7^. To validate the theoretical predictions, numerical results based on the model are compared with experimental results from pump-probe reflectometry measurements. Moreover, comparisons are made between the stepwise doping model (SDM) and the gradient doping model (GDM), which offer valuable insights into the validity of the GDM as well as its limitations.

## Theoretical model

**Figure 1:**
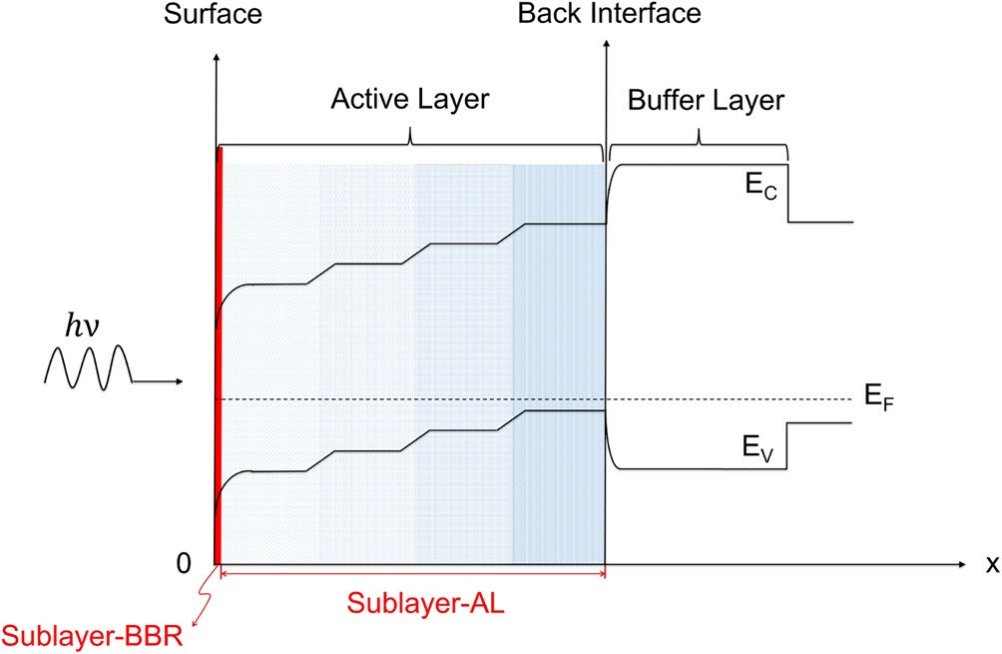
The band scheme of a 4-layer stepwise-doped GaAs photocathode with illustration of the two sublayers: the active layer (AL) and the band-bending region (BBR). Darker shading indicates higher doping concentrations.

Our theoretical analysis rests upon a “two-layer” concept, which has been experimentally verified in prior reports^5–7^. Specifically, we divide the active layer of a photocathode into two distinct sublayers based on the difference in electron behaviors: a thick active layer (AL), where most of photoelectron generation and transportation take place, and a very thin band-bending region (BBR) near the surface, where photoelectrons transported from the AL accumulate and become trapped. For simplicity, the sublayer AL is assumed to be loss-free, and all carrier decay processes are lumped into the sublayer BBR. Figure 1 illustrates the band scheme of a 4-layer stepwise-doped GaAs photocathode as well as the definitions of the two sublayers. At the surface of the photocathode, the conduction band and the valence band naturally bend downward as a result of the activation of the *p*-doped GaAs^8–10^. Downward band bending also occurs between adjacent doping layers due to their doping concentration difference, which forms a stepwise band profile in the AL, as shown in Fig. 1. Such a band profile creates strong, constant electric fields within narrow regions across the doping boundaries, while leaving the majority of each doping layer free of electric field^5,10^. Mathematically, the field distribution in the AL can be written as

1 $$\begin{aligned} E(x)= {\left\{ \begin{array}{ll} 0 &{} \text {within each doping layer} \\ \frac{\Delta E_F}{qW} &{} \text {on doping boundaries} \end{array}\right. } \end{aligned}$$

where $\Delta E_F$ is the difference between the quasi-Fermi levels of the adjacent doping layers, *q* is elementary charge, and *W* is the width of the downward bending region. *W* can be obtained through the relation^11,12^

2 $$\begin{aligned} W=\sqrt{\frac{2\varepsilon _0 \varepsilon _r \Delta E_F}{q^2 (N_h-N_l)}}, \end{aligned}$$

where $\varepsilon _0$ and $\varepsilon _r$ are the permittivity of vacuum and the relative permittivity of GaAs, respectively, and $N_h$ and $N_l$ are the higher and lower doping concentrations of the adjacent doping layers, respectively.

For each doping layer, the position of the quasi-Fermi level can be calculated through^13,14^

3 $$\begin{aligned} E_F = k_0 T \left( ln \frac{p}{N_V} + 0.35355 \frac{p}{N_V} \right) , \end{aligned}$$

where $k_0$ is the Boltzmann constant, *T* is temperature ($T = 300$ K is used in our model), *p* is the doping concentration, and $N_V$ is the effective density of states in the valence band. Note that, in heavily doped *p*-GaAs, the doping concentration *p* is usually comparable or even higher than the value of $N_V$, which is $7\times 10^{18}$ $\text {cm}^{-3}$ for GaAs. Therefore, the second term in Eq. (3) cannot be neglected as with non-degenerate semiconductors^15,16^. It is then straightforward to write the quasi-Fermi level difference between adjacent doping layers as

4 $$\begin{aligned} \Delta E_F = k_0 T \left( ln \frac{N_h}{N_l} + 0.35355\frac{N_h-N_l}{N_V}\right) . \end{aligned}$$

**Figure 2:**
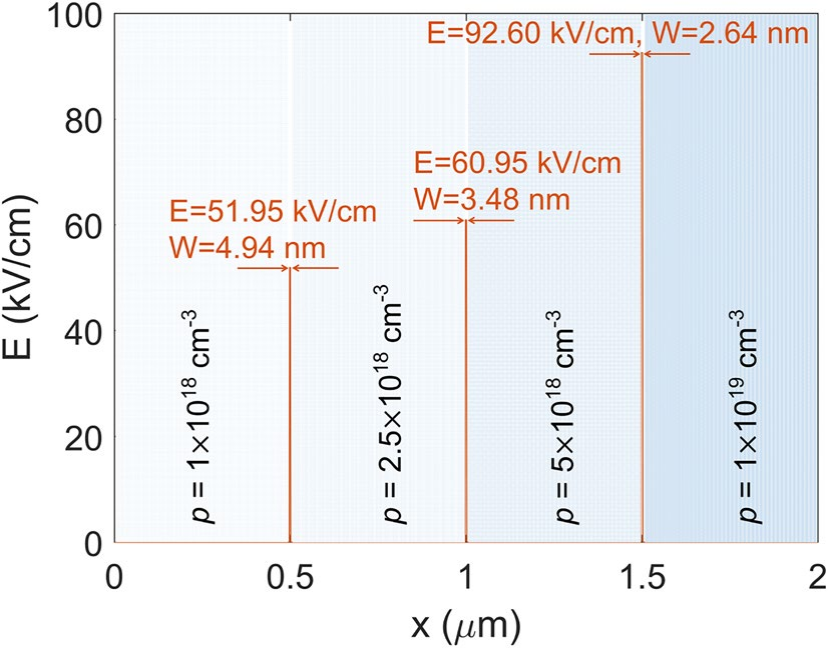
The electric fields generated inside a sample stepwise-doped structure. The lines between adjacent doping layers indicate the strengths of the electric fields in those regions. The widths *W* of these regions are also labeled.

Now, the electric field distribution in the AL can be determined by substituting Eqs. (2) and (4) into Eq. (1). An example of such a field distribution is shown in Fig. 2, where the doping parameters of an actual GaAs photocathode sample are given, along with the calculated values of *E* and *W* on each doping layer boundary.

In the sublayer AL, the governing equation is the simplified one-dimensional continuity equation^5^

5 $$\begin{aligned} \frac{\partial n(x,t)}{\partial t} = D \frac{\partial ^2 n(x,t)}{\partial x^2} + \mu _{n} \frac{\partial \left[ E(x)n(x,t) \right] }{\partial x}, \end{aligned}$$

where *n*(*x*, *t*) is the photoelectron concentration in the AL, *D* is electron diffusion coefficient, $\mu _n$ is electron mobility, and *E*(*x*) is the built-in electric field induced by the doping structure. Note that the *E*(*x*) here is given by Eq. (1), and $\mu _n$ and *D* are related through the Einstein relation $D/\mu _n=k_0T/q$. The initial condition for Eq. (5) is $n(x,0)=n_{0}e^{-\alpha x}$, where $\alpha$ is the absorption coefficient in the AL, and $n_0$ is a scale factor representing the initial photoelectron concentration on the device surface. Such an initial condition is the result of an instantaneous-excitation assumption, which is valid when the photoelectrons are generated via injection of femtosecond pulses^6^. For simplicity, $n_0$ has been chosen in our model so that $\int _{0}^{d}n_{0}\,e^{-\alpha x}dx=1$, here *d* is the thickness of the AL. A Dirichlet boundary condition $n(x=0,t)=0$ is applied to the AL-BBR interface, while a Neumann boundary condition ${\partial n(x=d,t)}/{\partial x}=0$ is applied to the back interface against the buffer layer.

**Figure 3:**
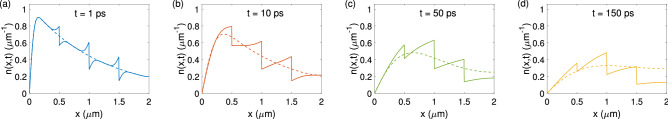
The distribution of electron concentration *n*(*x*, *t*) in the sublayer AL at different values of the time delay *t*, predicted by the SDM (solid) and the GDM (dashed) with a doping profile as shown in Fig. 2 and a diffusion coefficient $D=20$ $\text {cm}^{2}$/s.

According to Eqs. (1) and  (5), in a stepwise-doped photocathodes, electron diffusion exists throughout the entire AL, whereas electron drift only occurs on the boundaries between different doping layers. Using the device shown in Fig. 2 as an example and numerically solving the differential equation (5), the time evolution of the electron concentration *n*(*x*, *t*) inside the AL can be obtained. Figure 3a–d show the distribution of *n*(*x*, *t*) (solid lines) across the AL at four different delay times after the pulse excitation: 1 ps, 10 ps, 50 ps and 150 ps, respectively. Evidently, abrupt changes of the electron concentration are caused by the built-in electric fields on the boundaries between different doping layers. Such “sawtooth-shape” distributions are in stark contrast to the smooth *n*(*x*, *t*) profiles predicted by the GDM, as illustrated by the dashed lines in Fig. 3 for the same device^5^. When time delays are small, the GDM appears to result in fairly close predictions on the “average” trends of *n*(*x*, *t*), as shown in Fig. 3a,b. As time progresses, however, the discrepancies between the GDM and the SDM grow larger, as evident from Fig. 3c,d. For example, at the back interface ($x=2 \; \upmu \text {m}$), the electron concentration given by the GDM is 39% higher than that given by the SDM when $t=50$ ps (see Fig. 3c), and this difference grows to 56% when $t=150$ ps (see Fig. 3d).

**Figure 4:**
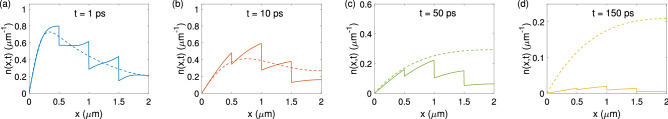
Similar comparisons between the SDM and the GDM as in Fig. 3 with $D=160$ $\text {cm}^{2}$/s.

The value of the diffusion coefficient *D* also appears to affect the accuracy of the GDM. There has been a wide range of *D* values reported in the literature for GaAs photocathodes, ranging from 20 $\text {cm}^{2}$/s to 160 $\text {cm}^{2}$/s^5,6,17–20^. The results shown in Fig. 3 are obtained with $D=20$ $\text {cm}^{2}$/s. If *D* is set to 160 $\text {cm}^{2}$/s, the difference between the SDM and the GDM is considerably more pronounced, as demonstrated in Fig. 4a–d.

**Figure 5:**
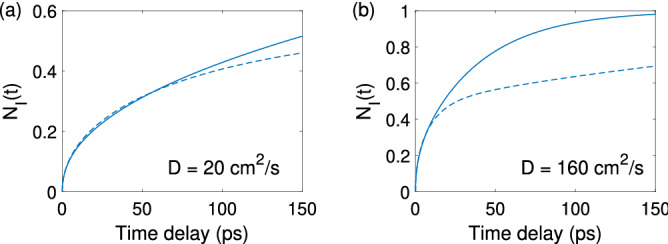
The total number of photoelectrons $N_I(t)$ transported from the AL into the BBR, predicted by the SDM (solid) and the GDM (dashed), with (**a**) $D=20$ $\text {cm}^{2}$/s and (**b**) $D=160$ $\text {cm}^{2}$/s.

Based on the above comparisons, it is evident that the SDM and the GDM in general predict different photoelectron distributions in the AL. Although the GDM is able to capture the average trend of the electron behaviors within small delay times, for devices with large diffusion coefficients or for cases involving long delay times, the GDM apparently tends to *underestimate* the rate of electron migration toward the BBR, which leads to noticeable errors. Such errors can be quantitatively assessed by studying the total number of photoelectrons $N_I(t)$ transported from the AL into the BBR, where $N_I(t)$ is defined as $N_I(t)=1- \int _{0}^{d} n(x,t) dx$. Figure 5a,b show the time evolution of $N_I(t)$ with both the SDM (solid) and the GDM (dashed) for $D=20$ $\text {cm}^{2}$/s and 160 $\text {cm}^{2}$/s, respectively. In both cases, the SDM predicts a faster overall AL-to-BBR injection rate than the GDM, indicating a trend of underestimation by the latter. To numerically gauge this error, we compare the two models when the SDM-predicted $N_I(t)$ reaches 0.5, i.e., when 50% of the total generated photoelectrons transport from the AL into the BBR. We find that, for both Fig. 5a,b, the GDM yields a prediction about 9.7% lower than the SDM.

## Theory-experiment comparisons

Given the different photoelectron behaviors predicted by the two models, it is interesting to investigate the impact of stepwise doping on the transient reflectivity change of actual devices. Previously, we have shown that pump-probe transient reflectivity (PPTR) measurements offer great insight into the dynamic behaviors of photoelectrons and can help determine key device parameters, such as diffusion coefficient and carrier lifetimes, if a proper theoretical model is used^5,7^. However, in these prior works, the GDM is used as the theoretical model. Here, we apply the SDM to the experimental data and seek to compare the outcome with results from the GDM.

The specific approach is quite similar to our previous reports (see, for example, Ref.^5^). Based on the Drude model, the transient change of surface reflectivity $\Delta R(t)$, which can be experimentally characterized through a PPTR measurement, is proportional to the device surface charge density *N*(*t*), which is described in our model by the electron population density in the BBR. The governing equation for electron population in the BBR can be written as

6 $$\begin{aligned} \frac{\partial N(t)}{\partial t} = J(t) - \varGamma N(t), \end{aligned}$$

where *J*(*t*) is the AL-to-BBR injection flux and $\varGamma$ is the electron decay rate. Note that *J*(*t*) is related to $N_I(t)$ through $J(t) = {dN_I(t)}/{dt}$, and $\varGamma$ combines all the effects that lead to the decay of the photoelectron population in the AL and the BBR. Numerically solving Eqs. (5) and (6) with an instantaneous initial excitation results in a solution of *N*(*t*) that represents the transient evolution of the surface charge density upon the injection of a femtosecond pulse. By comparing such a theoretical prediction with an experimental PPTR trace, key device parameters can be determined.

**Figure 6:**
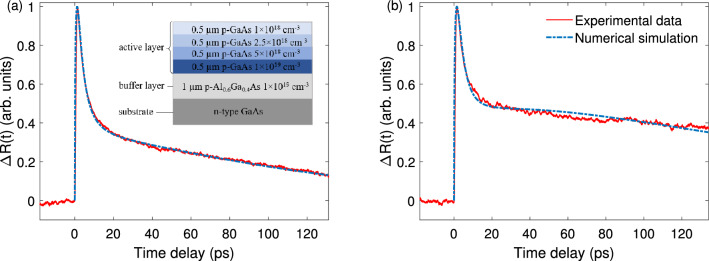
Theoretical predictions based on the SDM show excellent agreement with the experimentally measured PPTR traces for GaAs photocathode samples fabricated with (**a**) MOCVD and (**b**) MBE. Inset: The doping structure of the tested photocathodes.

The PPTR measurements are performed with a pump-probe reflectometer based on a 6.5-fs Ti:sapphire laser operating at a center wavelength of 800 nm and a repetition rate of 83 MHz. Details about the reflectometer can be found elsewhere^21^. Two stepwise-doped GaAs photocathodes have been tested, one fabricated with metal organic chemical vapor deposition (MOCVD) and the other fabricated with molecular-beam epitaxy (MBE). The two devices share the same doping structure, which is illustrated in Fig. 6a inset. The measured PPTR traces for the two devices are shown in Fig. 6a,b. Both traces are normalized to their peak values. A sharp rising edge is featured in both figures at $t=0$, which correspond to the injection of the pump pulse. A quick decay of the reflectivity is seen immediately following the peak, but it is quickly replaced by a slow, steady decay after about 10 ps.

The above general behaviors of the transient reflectivity bear the feature of a *bi-exponential* decay^5,6^. It is well known that multiple decay channels exist in GaAs photocathodes^20^. In our current model, the total electron population accumulated in the BBR experiences two independent decay processes, which cause the bi-exponential decay of the transient reflectivity. This can be incorporated in our model by first invoking the Drude model $\Delta R(t) \propto N(t)$ and then writing *N*(*t*) as $N(t)= C_1 N_{\varGamma 1}(t) + C_2 N_{\varGamma 2}(t)$, where $N_{\varGamma 1}$ and $N_{\varGamma 2}$ represent the populations of two separate groups of electrons. The two groups of electrons each independently follows Eq. (6) with their own decay rates of $\varGamma _1$ and $\varGamma _2$, respectively, and $C_1$ and $C_2$ are the corresponding weighting factors under the restraint of $C_1+C_2=1$. By setting proper values of the parameters $\varGamma _1$, $\varGamma _2$, $C_1$, $C_2$, and *D*, and solving Eq. (6), theoretical predictions of the transient reflectivity $\Delta R(t)$ can be made.

Such theoretical predictions based on the SDM have shown excellent agreement with the experimentally measured PPTR traces, as demonstrated in Fig. 6 for both the MOCVD and the MBE devices, provided that proper device parameters are chosen. This validates the effectiveness of the SDM. The corresponding device parameters used in the theoretical model are listed in Tables 1 and 2, respectively. To highlight the differences between the SDM and the GDM, Tables 1 and 2 also include the device parameters extracted from the GDM using the same devices^5^. Comparing the results from the two models, it is evident that, while the SDM and the GDM give similar assessments on the carrier decay parameters, the GDM consistently leads to greater values of the diffusion coefficient *D* than the SDM. This finding appears to support the conclusion made in the “Theoretical model” section, which suggests that the GDM tends to *underestimate* the rate of photoelectron transportation in a stepwise-doped device. As such, when the GDM is used to predict the experimental result based on a stepwise-doped device, a *greater-than-normal* value of *D* has to be used to compensate the underestimation of electron drift. From this point of view, it is reasonable to conclude that, while the GDM is capable of capturing the main characteristics of photoelectron transportation in a stepwise-doped photocathode, it does suffer the drawback of inaccurate assessment of the diffusion coefficient.

**Table 1:** Device parameters used in the theoretical computation for the MOCVD device in Fig. 6a.

Model type	*D* ($\text {cm}^{2}$/s)	$C_1$	$\tau _1$ (ps)	$C_2$	$\tau _2$ (ps)
Stepwise	110	0.9739	1.55	0.0279	80
Continuous	160	0.942	1.3	0.058	80

**Table 2:** Device parameters used in the theoretical computation for the MBE device in Fig. 6b.

Model type	*D* ($\text {cm}^{2}$/s)	$C_1$	$\tau _1$ (ps)	$C_2$	$\tau _2$ (ps)
Stepwise	120	0.9477	1.8	0.055	180
Continuous	160	0.911	1.5	0.089	180

## Discussion

### The assumption of a decay-free AL

**Figure 7:**
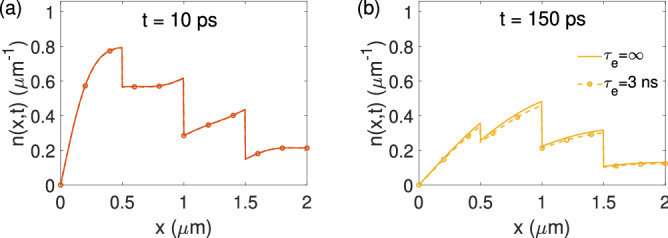
Impact of photoelectron decay in the AL on the distribution of *n*(*x*, *t*) at (**a**) $t=10$ ps and (**b**) $t=150$ ps, with $\tau _e=\infty$ (solid) and $\tau _e=3$ ns (dashed/markers).

So far, in our two-layer model, the sublayer AL has been assumed to be decay-free, as indicated by Eq. (5). In reality, however, carrier decay may exist in the AL. It is thus interesting to investigate how the inclusion of electron decay in the AL impacts the applicability of the two-layer model. To consider the decay, the governing continuity equation (5) is modified to

7 $$\begin{aligned} \frac{\partial n(x,t)}{\partial t} = D \frac{\partial ^2 n(x,t)}{\partial x^2} + \mu _{n} \frac{\partial \left[ E(x)n(x,t) \right] }{\partial x} - \frac{n(x,t)}{\tau _e}, \end{aligned}$$

where $\tau _e$ is electron lifetime in the AL. The impact of this new decay term can be qualitatively estimated via the following assessment. On one hand, prior research has shown that the lifetime of minority carrier in *p*-doped GaAs is generally above 1 ns^22,23^. On the other hand, for the devices studied here, the majority of the generated photoelectrons (>50%) are transported from the AL into the BBR within the first 150 ps, as shown in Fig. 5. Therefore, it is believed that the inclusion of electron decay in the AL should not significantly alter the photoelectron behaviors inside the AL. To verify this assessment, numerical simulations based on Eq. (7) have been carried out and the results are shown in Fig. 7. In Fig. 7a, the photoelectron concentration *n*(*x*, *t*) is calculated at a delay time of $t=10$ ps with $\tau _e=\infty$ (decay free) and 3 ns. In Fig. 7b, similar comparisons are made at a delay time of $t=150$ ps. In both cases, the difference introduced by the electron decay is negligible, which justifies the assumption of a decay-free AL in the current context. Note that $\tau _e=3$ ns has been chosen here as a representative value of the AL electron lifetime based on a recent study of GaAs with similar doping concentrations^23^.

### The Einstein relation

In the “Theoretical model” section, the Einstein relation $D=\mu _n k_0 T/q$ has been used to simplify the theoretical analysis based on Eq. (5). However, it has been shown that, when the doping concentration is comparable or even greater than the effective density of valance/conduction-band states, as in the current case, it is more accurate to use a *generalized* Einstein relation between *D* and $\mu _n$^13,14^, which takes the form of

8 $$\begin{aligned} D=\mu _n \frac{k_0T}{q} \left( 1+0.35355\frac{p}{N_V}\right) , \end{aligned}$$

where *p* is the doping concentration and $N_V$ is the effective density of states in the valence band. Under this generalized Einstein relation, the diffusion coefficient *D* is no longer a constant across the AL since it is now dependent upon the doping concentration. The continuity equation in the AL must be modified to

9 $$\begin{aligned} \frac{\partial n(x,t)}{\partial t} = D \frac{\partial ^2 n(x,t)}{\partial x^2} + \frac{\partial D}{\partial x}\frac{\partial n(x,t)}{\partial x} + \mu _{n} \frac{\partial \left[ E(x)n(x,t) \right] }{\partial x}, \end{aligned}$$

where an extra term proportional to $\partial D/\partial x$ is introduced.

**Figure 8:**
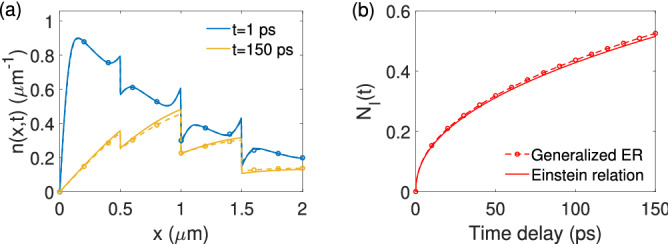
The effect caused by the generalized Einstein relation on (**a**) the electron distribution *n*(*x*, *t*) in the AL at different delay times and (**b**) the AL-to-BBR electron injection $N_I(t)$. Results obtained using the Einstein relation are represented by solid lines. Results obtained using the generalized Einstein relation are represented by dashed lines with circular markers.

The impact of this extra term in the current context is investigated using the doping configuration shown in Fig. 2 as an example, and the results are summarized in Fig. 8. In Fig. 8a, the photoelectron concentration *n*(*x*, *t*) in the AL is obtained with both the Einstein relation (solid) and the generalized Einstein relation (dashed lines with markers) for $t=1$ ps and $t=150$ ps. For small time delays, e.g., $t=1$ ps, the correction caused by the generalized Einstein relation has no apparent effect on the overall distribution of *n*(*x*, *t*). As the time delay increases, discrepancies begin to appear but nevertheless remain small. The overall effect of such discrepancies can be further evaluated by computing the BBR injection rate, which is shown in Fig. 8b, where the injection population $N_I(t)$ is plotted against the time delay for both versions of the Einstein relation. The generalized Einstein relation appears to result in a slight increase in $N_I(t)$, which is less than 2% across all delay times. Therefore, for the devices under study here, the original Einstein relation is considered a valid approximation. Finally, it is worth mentioning here that, because of the intimate relation between *D* and $\mu _n$ through the Einstein relation, the justified use of the Einstein relation also suggests that the electron mobility can be treated as a constant in our model.

## Conclusion

In conclusion, the photoelectron transport dynamics in stepwise-doped GaAs photocathodes is thoroughly investigated using the SDM, with a special focus on its improvement from the conventional GDM. By solving the one-dimensional continuity equation with a distinctive field distribution caused by the stepwise doping profile, the time-dependent electron concentration distribution in the AL is obtained. The resulted ultrafast evolution of the electron population in the BBR is compared with experimental PPTR measurements. Excellent agreements are achieved, and key device parameters, such as the diffusion coefficient and the electron decay lifetimes, are extracted. Comparisons with the GDM indicate that the continuous-doping treatment causes the photoelectron transport rate in the AL being underestimated in the theoretical model and the diffusion coefficient *D* being overestimated during theory-experiment comparisons. Finally, the validity of a decay-free AL and the suitability of the Einstein relation are examined and verified. The work establishes the importance and the effectiveness of the SDM, which offers a more accurate theoretical tool for analyzing III-V semiconductor photocathodes.

## Data Availability

The authors declare that the data supporting the findings of this study are available within the article. All other relevant data are available from the corresponding author upon reasonable request.
